# Characteristics of Populations of the Russian Federation over the Panel of Fifteen Loci Used for DNA Identification and in Forensic Medical Examination 

**Published:** 2011

**Authors:** V. A Stepanov, O.P. Balanovsky, A.V. Melnikov, A.Yu. Lash-Zavada, V.N. Khar’kov, T.V. Tyazhelova, V.L. Akhmetova, O.V. Zhukova, Yu.V. Shneider, I.N. Shil’nikova, S.A. Borinskaya, A.V. Marusin, M.G. Spiridonova, K.V. Simonova, I.Yu. Khitrinskaya, M.O. Radzhabov, A.G. Romanov, O.V. Shtygasheva, S.M. Koshel’, E.V. Balanovskaya, A.V. Rybakova, E.K. Khusnutdinova, V.P. Puzyrev, N.K. Yankovsky

**Affiliations:** Institute for Medical Genetics, Russian Academy of Medical Sciences; Vavilov Institute of General Genetics, Russian Academy of Sciences; Forensic Centre, Ministry of Interior of Russian Federation; Institute of Biochemistry and Genetics, Ufa Research Centre, Russian Academy of Sciences; Research Centre for Medical Genetics, Russian Academy of Sciences; Genome Diagnostics, Ltd.; Dagestan State University; Katanov Khakas State University; Geography Faculty, Lomonosov Moscow State University

**Keywords:** microsatellites, short tandem repeats, allelic frequencies, forensic medical examination, DNA identification, population of Russia, reference database, genetic diversity, gene geography

## Abstract

Seventeen population groups within the Russian Federation were characterized for the first time using a panel of 15 genetic markers that are used for DNA identification and in forensic medical examinations. The degree of polymorphism and population diversity of microsatellite loci within the Power Plex system (Promega) in Russian populations; the distribution of alleles and genotypes within the populations of six cities and 11 ethnic groups of the Russian Federation; the levels of intra- and interpopulation genetic differentiation of population; genetic relations between populations; and the identification and forensic medical characteristics of the system of markers under study were determined. Significant differences were revealed between the Russian populations and the U.S. reference base that was used recently in the forensic medical examination of the RF. A database of the allelic frequencies of 15 microsatellite loci that are used for DNA identification and forensic medical examination was created; the database has the potential of becoming the reference for performing forensic medical examinations in Russia. The spatial organization of genetic diversity over the panel of the STR markers that are used for DNA identification was revealed. It represents the general regularities of geographical clusterization of human populations over various types of genetic markers. The necessity to take into account a population’s genetic structure during forensic medical examinations and DNA identification of criminal suspects was substantiated.

## INTRODUCTION 

Molecular genetic analysis methods are now widely applied in the identification of the biological samples of individuals: victims of crimes, disasters, and acts of terrorism, criminals, and contingent of special divisions of armies or law enforcement. A genetic DNA analysis in forensic medical examinations has two stages. At the first stage, the DNA characteristics of the samples collected at the locus delicti are determined. At the second stage, they are matched with the DNA collected from the suspects or relatives of the victims. If there is no match of the genotypes, that points to the fact that the samples examined do not belong to the individual in question (taking into account the exclusion probability). When genotypes match, the probability of their random matching, i.e., the probability that other individuals may have the same genotypes, is also taken into account. 

The probability of a random match is calculated on the basis of data on the occurrence frequencies of the alleles (and genotypes) of the analyzed panel of genetic markers in reference populations. In order to create such reference databases, population samples collected with allowance for the population genetic structure of certain ethno-territorial groups are used. Allelic frequencies in various populations and groups have been published and presented in databases. These reference databases serve as a legally valid basis for forensic medical conclusions in interpreting the results of genotype comparisons. 

 The reliability and efficiency of DNA identification depends on two key factors: on the choice of the locus panel and the choice of the reference population. 

**Selection of the loci panel**

. The genetic markers that are used for forensic medical expertise should be highly polymorphic and should possess a high resolution capacity. Multiallelic (mostly consisting of 8–10 alleles) unlinked microsatellite markers – STR (Short Tandem Repeats) loci are considered to be the most efficient ones. However, different panels of these STR markers are used in different regions. 

In Europe, Interpol uses two standards of loci sets – ENFSI (the European Network of Forensic Science Institutes) and EDNAP (the European DNA Profiling Group), consisting of seven STR loci each. In 2005, an agreement on the unification of the loci systems used in Europe was signed. The ENFSI proposed six more markers as candidates to be included into the European standard set (ESS) [1]. In 2009, the ENFSI added five out of six candidate markers to its standard, thus broadening the European Standard panel ESS to 12 STR: *TH01, vWA, D18S51, D8S1179, D3S1358, FGA, D21S11, D1S1656, D2S441, D10S1248, D12S391 * и * D22S1405* . In 2010, the standard was approved by the European Union.

Starting in 1994, the CODIS (Сombined DNA Index System) system has been in use in the United States, its full format comprising 13 loci ( *D7S820, D13S317, CSF1PO, TPOX, D16S539, TH01, vWA,* *D5S818, D18S51, D8S1179, D3S1358, FGA, D21S1). * The CODIS and ENFSI systems have seven markers in common from the EDNAP/ENFSI primary standard.

In all the aforementioned systems (with the exception of the polymorphic autosomal STR loci,) another locus (amelogenin) is used, the size of its PCR fragments being different on the X and Y chromosomes, which allows for the determination of the sex of an individual by analyzing the DNA of a biological sample. 

When creating these systems, among the several tens of STR loci that had been tested, the most highly polymorphic ones within the majority of the examined populations were selected. For the convenience of genetic typing, the PowerPlex 16 system was designed, enabling the simultaneous amplification of 16 polymorphic loci in a single test tube, which considerably simplifies the analysis and reduces its cost. In addition to the amelogenin locus and the 13 loci from the CODIS system, this kit also comprises two highly polymorphic and easily readable pentanucleotide markers ( *PentaD and PentaE* ) [2].

On December 3, 2008, the Federal Law of the Russian Federation On State Genomic Registration in the Russian Federation was adopted. The law provides for the creation of the Federal database of genomic information under the Ministry of the Interior of the Russian Federation. Order of the Ministry of the Interior of the RF no. 70 dated February 10, 2006, is the official statutory act regulating the gene typing procedures for DNA identification; in the edition dated May 21, 2008, it establishes a set consisting of 12 STR markers and the amelogenin locus, which is totally identical to the American CODIS standard, as a mandatory set. 

**Selection of the reference population**

. In order to reliably compare genotypes in each case, the choice of the reference population should depend on the group that the individual who has left biological marks belongs to. In actual practice, the reference population is usually selected among the populations represented in the criminal databases which were studied using this panel of STR markers. 

The less the reference population represents the gene pool of a tested group, the more individuals within this group have alleles that are not in the reference database, which results in a considerable decrease in the discrimination capacity of the method. There are correlations between the number (percentage) of individuals who have alleles that are not in the reference population and the genetic distance between the reference population and the population under analysis [3].

The use of an inadequate reference group may result in a decrease in the total identification probability by several orders of magnitude. The situation can be improved by introducing corrections based on the maximum degree of genetic differences between subpopulations within a reference population (e.g., an ethnic group). In order to introduce such a correction, it is necessary to have information on the genetic differentiation between populations ( *F* st) with respect to the loci used for each specific group within each specific territory. This correction permits the replacement of alleles and genotypes that are unknown for the reference population by their calculated frequencies, with allowance made for the differentiation degree *F* st [4]. It is assumed that these calculated frequencies take into account the maximally possible differences between the unknown and reference populations.

Even if the group of an individual to whom the biological sample belongs is unknown, it can be identified with a certain probability, provided that there are population databases. Thus, when identifying the victims of the World Trade Centre terrorism act in New York, if the remains belonged to an unknown group, the probability was calculated using all four major American groups as reference points; the most conservative estimate was used as the final one [5]. After four years, 1,594 remains have been identified out of 2,749; 850 of those were identified only on the basis of data of a DNA analysis [5].

The criminal databases and criteria of comparison were developed with allowance for the genetic characteristics of ethno-territorial groups (e.g., see [4]) and are published in accordance with specific rules [6].

In the United States and Europe, a large massif of population has been characterized with respect to the loci used in forensic medical examinations. In other regions, several tens of population groups have been known to have been studied on the basis of panels of ENFSI, EDNAP, and CODIS genetic markers [7–14].

Data on the distribution of individual genetic markers from these panels in Russian populations has remained fragmentary [15–18]. In terms of interpretability of the data, Russia stands out upon DNA identification by its diverse mix of nationalities and vast geographical expanse. The considerable differences in the range of individual features of the genomes that are typical of various ethnic groups, in particular, the spatially remote ones, have been well known. Numerous population genetic studies of the Russian population performed using various systems of genetic markers, including mtDNA, the Y chromosome, and autosomal markers, have demonstrated that the range of interpopulation variability for different ethnic and territorial groups of the RF exceeds considerably the variability of the entire population of Europe [19–22]. However, because of the absence of systematic information on the RF population in terms of the marker panels that are commonly accepted in the world, the data on the frequencies of genetic characteristics in the population of the U.S. and Europe are used in practice for DNA identification in the RF, although whether these data can be applied to the RF population has not been assessed.

In this context, our work was aimed at determining the allelic frequencies of 15 autosomal STR loci from the PowerPlex 16 system in six urban population groups and 11 ethnic groups in the RF. A solution to this problem will allow to characterize the genetic variability of the Russian population using this system of markers and will lay the basis for the creation of our own reference population for DNA identification and forensic medical examinations in Russia. 

## EXPERIMENTAL 

**Populations**

Seventeen population groups with a total of 1,156 people representing different geographical regions of Russia (European part of the RF, the North Caucasus, the Volga–Ural region, Siberia) and belonging to different linguistic groups and different anthropological types were examined. 

Six samplings represent the Russian urban population: Moscow ( *N* = 60), Belgorod ( *N* = 50), Orel ( *N* = 51), Orenburg ( *N* = 50), Yaroslavl ( *N* = 50), and Tomsk ( *N* = 185). Eleven samplings represent a wide range of the Russian population and neighboring countries: Komi ( *N* = 50), Mari ( *N* = 52), Khakas ( *N* = 92), Bashkir ( *N* = 70), Tatar ( *N* = 61), Chuvash ( *N* = 53), Dargins ( *N* = 48), Avars ( *N* = 50), Lezgins ( *N* = 50), Ukrainians ( *N* = 138), and Belorussians ( *N* = 46).

**Molecular biology techniques**

The amplification of 15 STR loci and the sex marker (amelogenin gene) was carried out in the multiplex PCR format (one multiplex per all 16 loci) on Applied Biosystems and Biometra gradient amplifiers under the conditions that were recommended by the manufacturer of the commercial PowerPlex system (Promega). Fluorescently labeled PCR fragments were separated by capillary gel electrophoresis on an ABIPrism 3130 and an ABIPrism 310 genetic analyzer (Applied Biosystems). The genotypes were read using Gene Mapper software (Applied Biosystems). The quality of gene typing was controlled using the standard set of alleles of all 16 microsatellites (“ladder”) supplied within the PowerPlex 16 system; the “ladder” were loaded in each gene typing cycle (in each run). 

**Methods of statistical analysis of the results**

The data were analyzed using the modern statistical approaches employed in population genetics and forensic medicine. Correspondence of the observed genotype distributions to the Hardy–Weinberg equilibrium was estimated by the exact test of Guo and Thompson [23] implemented using the Arlequin and GenePop software. The genetic diversity of populations and the genetic variability of 15 STR were analyzed using the Arlequin software [24].

The genetic differentiation of the populations was analyzed by a calculation of pairwise *F* st values and by an analysis of molecular variance (AMOVA), using the matrix of root-mean-square discrepancies in repeat numbers of *R* st. The dendrogram illustrating the genetic relationships between the populations was constructed using the unweighed pair group method with the arithmetic mean (UPGMA) in PHYLIP software.

The variability of the studied loci in the population of North Eurasia was analyzed using the database on the frequencies of microsatellite markers in 51 populations that we compiled (the total sampling volume was 8,700 individuals). The database comprised both our own results presented in this paper and the data from earlier studies [25–39], including data on the populations of 12 countries (Belorussia, Bosnia, Greece, China, Macedonia, Mongolia, Pakistan, Poland, Russia, Slovakia, Sweden, and the Czech Republic). The database contains information on 17 loci ( *D3S1358, TH01, D21S11, D18S51, D13S317, D7S820, D16S539, CSF1PO, vWA, D8S1179, TPOX, FGA, D5S818, PentaD, PentaE, D2S1338, * and * D19S433* ). However, since five markers ( *D5S818, PentaD, PentaE, D2S1338, * and * D19S433) * had not been studied in a number of populations, the remaining 12 loci were used in the analysis.

The analysis of this vast massif was carried out using both statistical and cartographic gene-geographic. The statistical analysis consisted of the calculation of genetic distances according to Nei [40] using the DJgenetic software designed by Yu.A. Seregin and E.V. Balanovskaya. The Statistica 6.0 program (StatSoft. Inc., 2001) [41] was used to visualize the resulting genetic distance matrix on a multidimensional scaling diagram.

Heterozygosity with respect to each locus was calculated, and the averaged (over 12 loci) values of heterozygosity were obtained in each population. These values were mapped using GeneGeo software that was developed by a number of authors for several years. The calculation of interpolated heterozygosity values was performed on the basis of the data in reference points (immediately in the populations under study) to a uniform grid consisting of 335,661 nodes (881× 381); the 301,681 nodes remaining after the water area were eliminated. Interpolation was performed using the generalized Shepard’s method. The cube of the weighting function was employed; i.e., the contribution of each point into the calculated value in a certain node was in reverse proportion to the cube of the distance between the reference point and the node; the reference points at a distance of more than 3,000 km were not taken into account. 

The discrimination potential of the system, which consisted of 15 microsatellites, was estimated using standard medical forensic indices that included the matching probability (MP), power of discrimination (PD), power of exclusion (PE), and paternity index (PI) [42].

## RESULTS AND DISCUSSION 

**Genetic variability of 15 STR PowerPlex 16**

**Fig. 1:**
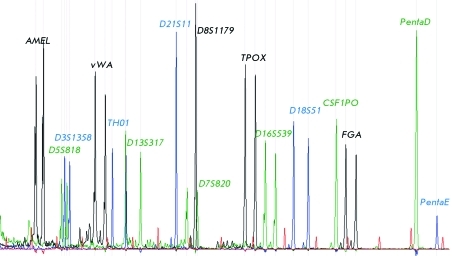
Multiplex genotyping of 15 microsatellites and the amelogenin locus ( *AMEL* ) in the PowerPlex 16 System by capillary electrophoresis.

In addition to 15 unlinked autosomal STR markers, the PowerPlex 16 system, which is intended for determining an individual’s genetic profile, comprises the marker of the amelogenin gene, which is located on X and Y chromosomes and is required for sex determination. *Fig. 1* shows an example of the multiplex gene typing of amelogenin and 15 satellites from the PowerPlex 16 system in one of the samples. Only the panel of microsatellite markers (15 STR) was used to perform the analysis in this study.

The results of a study of the genetic variability of these 15 STR in Russia and neighboring countries are listed in *Table 1* . The average level of intra-population genetic diversity (expected heterozygosity, He) of 15 STR in the populations under study was 0.796; the most variable loci (He > 0.85) – *D21S11, D18S51, PentaE* , and *FGA * – have more than 15 alleles. The highest number of alleles was found in loci *FGA* (20), *PentaE* (18), and *D18S51 * (18).

Pentanucleotide microsatellites *PentaE * are characterized by the highest dispersion of the repeat number (the 18-repeat difference between the shortest and the longest alleles) and *PentaD * (17-repear dispersion). The least polymorphic marker (He = 0.612), *TPOX* ,has eight alleles. The expected heterozygosity of the remaining 10 microsatellites of the PowerPlex 16 system varies within the range 0.74 < He < 0.82, the number of alleles detected varying from 8 to 12.

**Distribution of alleles and genotypes over populations**

In the populations consisting of 255 genotype distributions (15 loci in 17 samplings) that were studied, the deviation from the Hardy–Weinberg equilibrium (HWE) ( *p * < 0.05) was detected only in 21 of them. The accumulation of deviations from the Hardy–Weinberg equilibrium was detected only in the Tomsk population (five loci out of 15). However, when introducing the Bonferroni correction for comparison multiplicity, the actual significance level for the kit consisting of 15 tests per population is equal to 0.0035; therefore, with allowance for the Bonferroni correction, only one deviation from the Hardy–Weinberg equilibrium ( *FGA* locus in the Tomsk population) turned out to be statistically significant.

**Table 1:** Genetic variability of 15 STR from the PowerPlex 16 system

Locus	Expected heterozygosity	Average expected heterozygosity per population	Number of alleles	Average number of alleles per population	Dispersion of repeat numbers	Average dispersion of repeat numbers per population
*D3S1358*	0.77543	0.76634	8	5.647	7	4.765
*TH01*	0.78141	0.76693	8	5.588	6	3.588
*D21S11*	0.84974	0.84229	17	10.588	9	6.353
*D18S51*	0.87419	0.86735	18	11.882	16	11.118
*PentaE*	0.91497	0.90474	18	15.176	18	16.118
*D5S818*	0.73859	0.73546	9	6.529	8	5.941
*D13S317*	0.79676	0.78925	10	7.176	9	6.176
*D7S820*	0.80174	0.79478	12	7.471	10	6.471
*D16S539*	0.78966	0.78064	9	7.000	7	5.941
*CSF1PO*	0.73503	0.73035	8	5.882	7	5.059
*PentaD*	0.82446	0.82034	13	8.588	17	8.588
*vWA*	0.79355	0.79053	10	7.176	9	6.235
*D8S1179*	0.79676	0.79044	11	8.471	10	7.647
*TPOX*	0.61227	0.60398	8	5.294	7	4.412
*FGA*	0.85811	0.85062	20	10.941	13	8.882
Average per locus	0.79618	0.78893	11.933	8.227	10.200	7.153

**Fig. 2:**
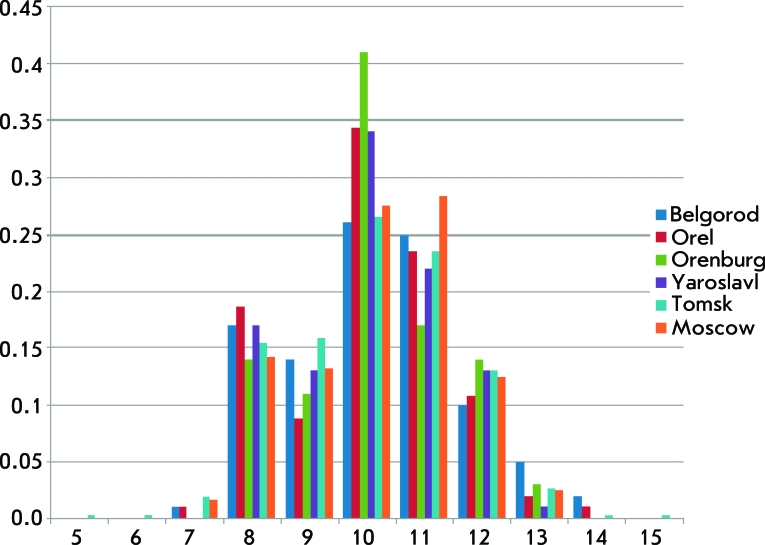
Allelic distribution of the D7S20 locus in populations of six Russian cities. The X axis shows the alleles (repeat numbers), the Y axis shows the allelic frequencies (fractions of one).

An example of allele distribution in populations ( *D7S820 * locus in six Russian cities) is shown in *Fig. 2* . The genetic variability indices of 17 populations over 15 microsatellites are summarized in *Table 2* . All 17 populations have close degrees of genetic diversity (the average heterozygosity fluctuating within 0.771–0.803). The highest degree of genetic variability was revealed in the populations of Lezgins, Ukrainians, and Tomsk residents; the lowest degree was revealed in the Mari, Khakas, and Orel residents.

**Gene geography of genetic diversity of PowerPlex 16 markers in North Eurasia**

**Fig. 3:**
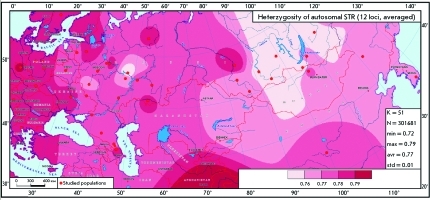
Map of average heterozygosity for 12 autosomal microsatellites ( *D3S1358, TH01, D21S11, D18S51, D13S317, D7S820, D16S539, CSF1PO, VWA, D8S1179, TPOX, * and * FGA* ). The color saturation level corresponds to the level of average heterozygosity (exact delimiter values are indicated on the map scale). Populations are depicted by red dots. In the legend window, the following parameters are indicated: the number of reference points (K); the number of the map grid nodes (N); minimum (min), maximum (max) and average (aver) heterozygosity values, and the standard deviation (std).

The heterozygosity of 12 loci ( *D3S1358, TH01, D21S11, D18S51, D13S317, D7S820, D16S539, CSF1PO, vWA, D8S1179, TPOX, * and *FGA) * was calculated in 51 populations of Russia and neighboring countries using both our data and the results obtained by other authors represented in the database compiled by us ( *Fig. 3* ). Although all the markers contained in the PowerPlex 16 panel were selected on the basis of the maximum intra-population variability (including heterozygosity), the populations in different Russian regions differ in terms of their heterozygosity level. The map demonstrates that the maximum heterozygosity (above 79%) is observed in the populations of Western and Central Europe and in the neighboring western regions of NIS countries. The heterozygosity level decreases gradually when moving eastwards. Thus, in the European part of Russia and the Trans-Urals, it is equal to 78%; in Central Asia and Altai, approximately 77%; in the Baikal region, less than 77%. This regularity of gradual decrease in heterozygosity across the entire Eurasian continent (from the Atlantic to the Pacific coast) can be clearly traced to an appreciable degree, although separate populations may fall out of the general trend (e.g., heterozygosity in the Kostroma population abruptly decreases). In the deep south, an increase in heterozygosity to maximum values exemplified by the Pakistani population was observed.

**Table 2:** Genetic variability (expected heterozygosity) of 17 studied populations with respect to 15 STR from the PowerPlex 16 system

Locus	Belgorod	Orel	Orenburg	Yaroslavl	Belorussians	Ukrainians	Komi	Mari	Tomsk	Khakas	Moscow	Dargins	Lezgins	Avars	Bashkir	Tatar	Chuvash
*D3S1358*	0.79434	0.77946	0.77232	0.75394	0.73459	0.79831	0.76929	0.78771	0.78909	0.68603	0.79342	0.77325	0.79535	0.77616	0.72816	0.72917	0.76712
*TH01*	0.76465	0.76024	0.78586	0.76707	0.78094	0.77249	0.74990	0.72087	0.78202	0.74157	0.75644	0.79518	0.74040	0.78384	0.78479	0.77686	0.77466
*D21S11*	0.83960	0.83460	0.85556	0.86465	0.86742	0.86777	0.85354	0.84055	0.85443	0.81528	0.85224	0.83224	0.86465	0.82020	0.79486	0.81222	0.84906
*D18S51*	0.87859	0.86469	0.87859	0.87071	0.86359	0.86551	0.87091	0.84839	0.87784	0.81938	0.88683	0.87149	0.86101	0.87273	0.86608	0.87847	0.87008
*PentaE*	0.90626	0.90390	0.90081	0.89778	0.89489	0.89926	0.90586	0.90497	0.91035	0.93258	0.90168	0.90548	0.91172	0.84869	0.92415	0.91695	0.91518
*D5S818*	0.73737	0.72898	0.73172	0.74404	0.74439	0.72419	0.73980	0.75243	0.75267	0.75956	0.71346	0.75022	0.76283	0.74465	0.69681	0.70383	0.71590
*D13S317*	0.76808	0.79616	0.80889	0.79434	0.81510	0.78100	0.78828	0.80284	0.80313	0.81332	0.77184	0.69825	0.77354	0.79960	0.79589	0.82834	0.77862
*D7S820*	0.81657	0.77985	0.75838	0.78101	0.78882	0.80208	0.80727	0.77072	0.80964	0.80928	0.79636	0.79232	0.81071	0.77273	0.81470	0.79230	0.80845
*D16S539*	0.70889	0.76471	0.76626	0.77455	0.77520	0.75109	0.76485	0.77857	0.76974	0.79615	0.77691	0.82456	0.79091	0.80444	0.81048	0.80734	0.80629
*CSF1PO*	0.73838	0.70938	0.75636	0.72970	0.73865	0.74740	0.76141	0.70874	0.73273	0.74584	0.71527	0.69737	0.67495	0.73172	0.72528	0.76440	0.73836
*PentaD*	0.82202	0.82392	0.81111	0.80869	0.83516	0.82482	0.81192	0.79593	0.83174	0.81682	0.81597	0.80855	0.85010	0.80000	0.82713	0.84677	0.81509
*vWA*	0.81212	0.77888	0.81960	0.80909	0.79312	0.80398	0.81818	0.76176	0.77623	0.76734	0.81653	0.75504	0.82525	0.81111	0.76053	0.75559	0.77466
*D8S1179*	0.74889	0.80606	0.79919	0.81899	0.80029	0.79702	0.81333	0.74571	0.79657	0.74964	0.79566	0.79298	0.79333	0.76545	0.81357	0.81358	0.78724
*TPOX*	0.64646	0.54533	0.61818	0.59879	0.55638	0.63578	0.60061	0.52502	0.62928	0.61469	0.59608	0.65175	0.74727	0.57354	0.60113	0.58231	0.54501
*FGA*	0.86586	0.81965	0.85131	0.85778	0.86168	0.85447	0.85960	0.82207	0.85388	0.83904	0.87171	0.80899	0.84909	0.84727	0.86701	0.87170	0.85948
Average per locus	0.78987	0.77972	0.79428	0.79141	0.79001	0.79501	0.79432	0.77109	0.79796	0.78043	0.79069	0.78385	0.80341	0.78347	0.78737	0.79199	0.78701

The longitude tendency towards decreasing geterozygosity (from the west to the east of North Eurasia) that was first described in the markers of the “criminal panel” has been well-known on the basis of the conventional gene-geographic studies of the population of the USSR. Heterozygosity maps of the conventional (immunobiochemical) markers are given in [43], which also demonstrate a decrease in variability from the European part towards Siberia. The reason for the decrease in heterozygosity can be accounted for by the more intense genetic drift in the relatively small and isolated Siberian populations; whereas the effect of genetic drift in the East, and particularly in the Western part of Europe, is levelled by intense population migrations.

**Genetic differentiation of populations**

**Table 3:** Comparison of 15 STR frequencies for each locus in Russian urban populations and in Caucasian Americans

Locus	*F*st	*p*
D3S1358	0.00169	0.02444+-0.00383
TH01	0.00238	0.00782+-0.00343
D21S11	0.00113	0.04008+-0.00603
D18S51	0.00380	0.00000+-0.00000
PentaE	0.00181	0.00196+-0.00136
D5S818	0.00091	0.13001+-0.01025
D13S317	0.00638	0.00000+-0.00000
D7S820	0.00436	0.00000+-0.00000
D16S539	0.00132	0.05963+-0.00636
CSF1PO	0.00205	0.02835+-0.00465
PentaD	0.00460	0.00000+-0.00000
vWA	0.00154	0.02639+-0.00540
D8S1179	0.00256	0.00391+-0.00185
TPOX	0.00438	0.00489+-0.00203
FGA	0.00095	0.06256+-0.00769

The analysis of the genetic differences between populations was performed by the molecular variance method (AMOVA) with account for the variation in allelic frequencies, and the dispersion of tandem repeat numbers revealed significant genetic variations between the groups of populations studied. All Russian populations, the Ukrainians, Belorussians, and Komis are characterized by a community of the gene pool with respect to the studied markers and the absence of a significant inter-population differentiation ( *F* st values compared pairwise are not higher than 0.25%). Meanwhile, the group of Slavic populations significantly differs from most of the other populations. Populations of the Volga-Ural region (the Tatars and Chuvash), as well as the Mari, have no significant genetic difference between each other; however, they differ from other ethnic groups. Two other groups that are characterized by significant differences from all the other groups are the populations from the North Caucasus (Dargins, Avars, and Lezgins), the Bashkirs, and the Khakas.

The total level of genetic differentiation of the pool consisting of 17 populations turned out to be relatively high ( *F* st = 0.0267, or 2.67%) and highly significant ( *p*  > 0.00001).

Meanwhile, the analysis inside the massif consisting of six Russian urban populations, in spite of the considerable territorial sparseness of the cities that represent the center of the European section of Russia (Moscow), its south (Belgorod, Orel), north (Yaroslavl), the Urals (Orenburg), and Siberia (Tomsk) revealed the total absence of inter-population differences between 15 microsatellites in these populations in terms of frequencies and molecular dispersion. The *F* st value in six Russian urban populations was equal to 0.00095 ( *p*  = 0.6187).

Within the context of using and studying 15 STR to perform DNA identification, these data point to the possibility of using the sum frequencies over Russian megapolises when carrying out a medical forensic expertise of the urban (predominantly Russian) population. In addition, these data indicate the necessity for accounting for data on the frequencies of the “identification” markers in other ethnic groups of the Russian Federation to perform calculations in these populations. 

The comparison of the frequencies of 15 STR in an aggregate sampling of the populations of Russian cities with the frequencies in Caucasian Americans supplied by Promega company [2] as the reference frequencies for the PowerPlex 16 system by an analysis of the molecular dispersion for each locus revealed reliable differences in frequencies in 12 out of the 15 microsatellite loci ( *Table 3* ).

**Genetic relationships between populations: phylogenetic analysis**

**Fig. 4:**
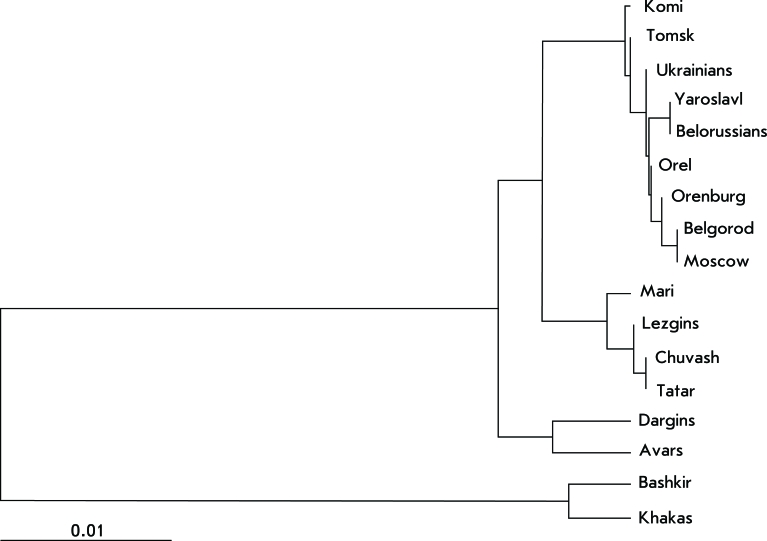
Dendrogram of the genetic distances between populations.

The tree of genetic inter-population relationships was constructed based on the matrix of pairwise genetic distances between populations with respect to the combination of the 15 STR loci obtained by AMOVA and with account for the differences in allelic frequencies and the dispersion of the tandem repeat number. The dendrogram constructed by the unweighed pair-group method with an arithmetic mean (UPGMA) in PHYLIP software is shown in *Fig. 4* .

The arrangement of populations on the dendrogram completely coincides with the revealed pattern of genetic differentiation in the Russian population over the DNA markers that are used for medical forensic expertise. The populations studied are grouped into four clusters, each of these clusters being characterized by a community of the gene pool of populations inside the cluster and significant differences (and large genetic distances) from the populations belonging to other clusters. 

The most distant cluster is formed by the Khakas and Bashkir populations, the ones with the highest proportion of the mongoloid race component. The rest of the populations are much closer to each other than they are to the Bashkirs or Khakas; however, they can also be clearly divided into three separate groups – Slavic populations (all of the Russian megapolises that were studied, Ukrainians, Belorussians) and Komis; populations of the Volga-Ural region (Chuvash, Tatar, Mari); and North Caucasus populations (Dargins and Avars). The location of the Lezgin population in a cluster with the Turk-lingual and the Uralian-lingual populations of the Volga-Ural region is a surprise. This is likely associated with random effects due to the small number of samplings. 

**Genetic relationships between populations: multidimensional scaling**

**Fig. 5:**
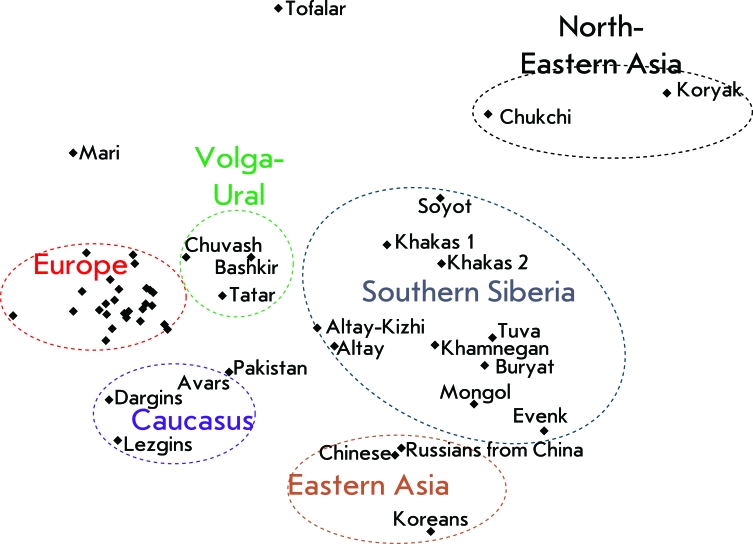
Location of the first two dimensions of the multidimensional scaling of the genetic distance matrix for 51 populations (17 populations from this paper and 34 populations from earlier published data). The European cluster includes the following populations: Swedes, Greeks, Komis, Czechs, Slovaks (2 samples), Polish (3 samples), Ukrainians, Belorussians, Russians from Belgorod (2 samples), Orel (2 samples), Yaroslavl, Kostroma, Novgorod, Pskov, Mineralnye Vody, Rostov-on-Don, Saratov, Orenburg, and Tomsk.

The location of the populations in the space within the first two dimensions of multidimensional scaling is shown in *Fig. 5* . The spatial distribution of a population represents the degree of similarity between the individual gene pools in the best way. It can be seen that all European populations are concentrated in the left-hand side of the plot. The proximity of populations in the European cluster points to the unity of the gene pool of the populations studied (the Swedes, the Greeks, the Poles, the Slovaks, a number of East Slavic populations, and the Komi). Two more clusters are located in direct proximity: the population of the Volga-Ural region and the North Caucasus population. It is noteworthy that, as opposed to a phylogenetic analysis, the multidimensional scaling places the Lezgins into a cluster together with the Dargins and Avars. The Asian populations are located in the right-hand side of the plot. Here, the largest and vastest (i.e. genetically diverse) cluster was formed by the South Siberian and Central Asian populations. The populations of the extreme northwest of Siberia (the Koryak and Chukchi) stand apart and form their own cluster. Finally, the East Asian populations (the Chinese and Koreans) also form a separate cluster. It is interesting that the population of Russians who have been living in China for several generations [35] cannot be genetically differentiated from the native populations of East Asia.

A conclusion can be reasonably drawn that the panel consisting of the 12 autosomal microsatellite loci that are used in the practical activity of medical forensic experts also happens to be highly informative for fundamental studies into the gene pool. Firstly, this fact is attested to by the consistency between the genetic clusterization of populations with respect to the markers set and the geographic (and linguistic) grouping of the same populations. Secondly, the distribution of populations within the plot space repeats their spatial distribution on the geographical map (e.g., the Koryaks and Chukchis are located in the top-right corner of the plot and in the top-right corner of the geographical map of Russia). Thirdly, the relative dimensions of the clusters correlate well with the concepts that were earlier formulated in science (e.g., decreasing heterozygosity when moving eastwards, the pronounced heterozigosity of the Siberian cluster). Let us specify that high interpopulation variability in Siberia agrees well with the low interpopulation variability (heterozygosity) of these populations ( *Fig. 3* ), since both features typically result from genetic drift, its intensity being higher in small and isolated Siberian populations. Another significant conclusion consists in the contrast between the homogeneity of European populations (such geographically distant from each other populations as the Swedes, Greeks, and Russians are almost indiscernible in the plot) and heterogeneity of the other regions studied. The populations of the Caucasus, Volga-Ural region, Southern Siberia, Northeastern Siberia, and East Asia have drastically different allelic frequencies. In addition, Siberian populations differ considerably between each other.

This fact is likely to be of great practical significance, since it becomes clear in which geographic range the databases concerning the frequencies of the markers used in medical forensic expertise can be employed. Thus, the use of separate databases for native populations of the Caucasus, the Volga-Ural region, and Siberia can be recommended when calculating the probability of a random match during the identification of a person. For Siberia, an even more detailed zoning may be required. The data on the allelic frequencies in Russian populations that are presented in our study may serve as one of the most important sources of information in the compilation of these databases. 

**Identification potential of 15 STR in populations of Russia and neighboring countries**

To assess the possibility of using the marker system under study for DNA identification in medical forensic expertise, the standard population statistic indices characterizing the identification potential of the marker system were determined. These indices include the matching probability (MP), the power of discrimination (PD), the power of exclusion (PE), and the paternity index (PI). The MP and PD indices are used in the DNA identification of a person, whereas the PE and PI indices are calculated when determining paternity. The values of these indices in certain populations, as well as those in Caucasian Americans, are listed in *Table 4* .

In general, all the populations studied had very high values of the discrimination potential of the PowerPlex 16 system. The matching probability of genotypes with respect to 15 STR markers for a total sampling of the Russian urban population was equal to 1 out of 281 000 000 000 000 000 (1 out of 281 quadrillion) individuals. In other populations, this index is slightly lower, but it still considerably exceeds all reasonable thresholds for DNA identification. 

It should also be noted that the paternity indices in all populations are higher than the values set by the statutory acts prevailing on the territory of the Russian Federation by several orders of magnitude. Thus, the following levels of evidence of the expert investigation was ascertained in Section 7 of the Instruction for Organization and Production of Expert Investigations in the Bureau of Medical Forensic Expertise approved by Order of the Ministry of Healthcare of the Russian Federation no. 161 dated March 24, 2003: The Bayesian probability of paternity is at least 0.9990, and the paternity index (PI) is at least 1,000 for a case of complete trio mother–child–putative father; and the Bayesian probability is at least 0.9975, and the PI is at least 400 for a duet child–putative father. 

The resulting indices of informativeness of the15 STR for DNA identification in a medical forensic expertise for the Russian and Ukrainian populations either exceed those of U.S. Caucasian Americans or are very close to them (the reference data provided by Promega company). In other ethnic groups of Russia, these indices are somewhat lower either due to the smaller volume of the samplings or due to the features of their population-genetic structure, but in any case they remain very highly informative. 

**RusDNAid DNA Identification Database**

**Table 4:** Identification parameters of 15 STR in certain populations

Population	Probability of genotype matching (MP)	MP recalculated for 1 out of...	Power of exclusion (PE)	Paternity index (PI)
Belgorod	1.66 x 10^-16^	6.33 x 10^15^	0.999998	742717
Orel	2.53 x 10^-16^	3.95 x 10^15^	0.9999992	1003109
Orenburg	1.06 x 10^-16^	9.36 x 10^15^	0.9999991	1065170
Yaroslavl	2.46 x 10^-16^	4.04 x 10^15^	0.9999997	3378695
Tomsk	3.44 x 10^-18^	2.81 x 10^17^	0.9999990	880293
				
Russians, in total	3.19 x 10^-18^	3.12 x 10^17^	0.9999989	834233
				
Belorussians	9.11 x 10^-17^	1.08 x 10^16^	0.999997	284297
Ukrainians	6.34 x 10^-18^	1.56 x 10^17^	0.9999995	1834277
Komi	5.60 x 10^-17^	1.73 x 10^16^	0.999998	451441
Mari	3.28 x 10^-16^	3.04 x 10^15^	0.99998	46918
Khakas	7.42 x 10^-17^	1.37 x 10^16^	0.99991	192783
				
Caucasian Americans		1.83 x 10^17^	0.9999994	1520000

Primary data on the allelic frequencies of 15 microsatellite loci from the PowerPlex 16 system in 17 populations within Russia and neighboring countries are represented in the RusDNAid database designed by us. The database is hosted online on the websites of the Institute of Medical Genetics, Siberian Branch of the Russian Academy of Medical Sciences (http://www.medgenetics.ru/web-resources/pp16-rus/) and the Vavilov Institute of General Genetics, Russian Academy of Sciences (www.vigg.ru/info/data_bases/human/DNAid). The frequencies mentioned can be used as reference frequencies (for the corresponding population or ethnic group) in order to calculate identification probabilities for a genetic expertise, including the identification of a person, establishment of paternity, etc. Moreover, these data can be used in comparative population genetic studies. 

## CONCLUSIONS 

Estimates of the genetic variability of microsatellite loci, which are used for DNA identifications that comply with the international standards for such studies, were obtained for the first time in this study for the population of Russia and neighboring countries. The informativeness and resolution capacity of the full panel of STR loci was first estimated, and the reference allelic frequencies for Russian urban populations, certain ethnic groups of the Russian Federation, and neighboring countries were obtained. 

The identification indices of the systems used for DNA identification based on the CODIS international standard comprising 13 STR loci, or its extended version consisting of 15 STR, that make up the PowerPlex 16 system (the reference allelic frequencies and indices of the identification capacity of gene typing systems) were estimated for most of the populations of European countries, the USA, Japan, and a number of other countries. These reference databases underlie the performance of the national services of medical forensic expertise. In Russia, until recently, there was no reference database on the locus contained in the standard identification panels. The results of the present study allow to fill this gap and offer a possibility to align the standards of personality typing with international practice. 

The spatial organization of genetic diversity, which was revealed by a gene-geographic method, phylogenetic analysis and multidimensional scaling on the basis of the panel of STR markers used for DNA identification, demonstrates the general regularity of the geographic clusterization of human populations on the basis of different types of genetic markers, from the conventional protein polymorphism to full-genomic SNP sets (e.g., see [22]); it shows a considerable tendency within the gene pool of the Russian population and neighboring countries towards subdivision and the necessity to account for a population’s genetic structure when performing medical forensic investigations and the DNA identification of persons in criminal cases.
